# Nursing master students’ experiences of and reflections on patient safety issues – a mixed-methods study using ecological momentary assessment

**DOI:** 10.1186/s12909-025-08281-6

**Published:** 2025-11-22

**Authors:** Marit Vassbotten Olsen, Ann-Chatrin Linquist Leonardsen, Monica Wammen Nortvedt, Roy Miodini Nilsen, Klas Karlgren

**Affiliations:** 1 Western Norway University of Applied Sciences, Inndalsveien 28, Bergen, 5063 Norway; 2 Østfold University College, Postal box code 700, Halden, 1757 Norway; 3 University of South-Eastern Norway, Raveien 215, Borre, 3184 Norway; 4 Karolinska Institutet, Stockholm, 171 77 Sweden

**Keywords:** Adverse events, Clinical practice, Ecological momentary assessment, Patient safety, Resilience

## Abstract

**Background:**

Patient harm is a serious problem within healthcare services. New methods and technologies improve possibilities to capture students’ experiences in clinical practice. Investigating nursing master students’ experiences may provide important insights about patient safety issues and positive benefits in education. The aim of this study was to explore nursing master students’ experiences and reflections on patient safety issues and adverse events during and after clinical practices.

**Methods:**

A mixed-methods convergent design was used, employing Ecological Momentary Assessment (EMA) combining ratings of six patient safety statements (Likert scales, 1 = totally disagree, to 5 = totally agree) with free-text reflections during clinical practice, and interviews after. Analyses of the quantitative data were combined with a thematic analysis of the qualitative data. In total, 16 nursing master students in nurse anaesthesia (*n* = 9), critical care nursing (*n* = 5) and operating room nursing (*n* = 2) participated in EMA during a 14-day period. In total, 105 EMA responses were obtained. All the students were invited to participate in the interviews afterwards, and 10 of them accepted.

**Results:**

The quantitative data indicated that, overall, the students considered their clinical practice as patient safe. Mean responses to the six statements were: *Observed* (Mean 2.5), *Discussed* (4.6), *Avoided* (2.5), *Experienced* (2.0), *Safe work* (4.6), *Threatened* (1.4). The participants, however, also provided accounts of events jeopardizing patient safety. Analysis of the students’ reflections during clinical practice resulted in three themes: 1) *Adverse events or near misses*, 2) *Communication and interactions in the team* and 3) *Using security procedures*. Post-practice interviews indicated that the EMA led to increased awareness of patient safety issues**.**

**Conclusions:**

Overall, few adverse events and near misses were observed. However, our results indicated that such events do occur and thus need to be addressed. The participants reported that engaging in the daily data collection increased their awareness of adverse events, near-misses and things that go well. The EMA method could be useful as a patient safety reflection tool for master nursing students and other healthcare students.

**Supplementary Information:**

The online version contains supplementary material available at 10.1186/s12909-025-08281-6.

## Background

The World Health Organization (WHO) states that around one in ten patients are harmed in healthcare, and more than three million deaths occur every year due to unsafe care [1]. The main adverse events reported are related to surgery, postoperative complications, drugs, nosocomial infections and obstetrics [2]. Various studies have contributed by researching patient safety issues in advanced clinical environments, such as failing to use the Safe Surgery Checklist (SSC), lack of adequate teamwork in operating theatres and emergency care units, as well as communication failures in patient transfer between operation theatres and postoperative anesthesia care units [3–6]. The rate of adverse events may be explained by the complexity of the patients, treatments and the organization itself. Hence, exploring practice errors as well as improving safety strategies such as safety-related education and training are important when aiming to reduce these incidents [7].

The theory–practice gap is well documented in healthcare in general, and in nursing specifically [8]. More discussion and research are recommended for covering what students need to be prepared for, when further learning in a work context [9]. Innovative learning methods are needed, and collaboration between clinical practice and education needs to be developed [10]. It is important to let students share their own experiences from clinical practice. These reflections have been shown to be positive for focusing on patient safety and learning from adverse events [11].

In Norway, there are national guidelines for the nurse anesthesia (NA), critical care nursing (CCN) and operating room nursing (ORN) master programs. These master programs consist of a combination of theoretical themes and 30 weeks of clinical practice, distributed over three to four periods. As a team member in intensive care units (ICU) or in operating rooms (OR), NA, CCN and ORN master students need to be encultured into a patient safety culture as well as gain adequate knowledge and competence about identifying and managing adverse events [12–14].

Most studies about nursing students’ clinical practice rely on the retrospective data collected using surveys, interviews or focus groups. Such methods are relatively undemanding to carry out, but they may suffer from recall bias, social desirability bias and other forms of distortions. Therefore, it may be preferable to study students’ experiences in their natural clinical context [15]. During the past couple of decades, Ecological Momentary Assessment (EMA) methods have become more established. Two other terms used for this are Experience Sampling Method (ESM) and Contextual Activity Sampling. They have their origins in diary methods and focus on investigating behaviors in their natural conditions, by longitudinally collecting information about behaviors, thoughts and feelings using ordinary smartphones, for example [16–18]. EMA offers a variety of possibilities concerning methodology, use of technology, data collection, analysis and reporting [19–22]. An advantage is that such methods eliminate retrospective memory biases and ensure ecological validity as data collection takes place in the natural context. Lachmann et al. [17], found an EMA approach suitable for collecting contextualized data in a clinical learning environment and concluded that they were able to collect data about students’ experiences on a level of detail that would not have been possible to achieve through conventional methods such as post-course questionaries or interviews. Similarly, Karlgren et al. [23], found striking differences in what nursing students reported about their experiences of competence and challenge during clinical placements compared to what the same students reported in interviews afterwards. However, EMA has not been widely used to capture learning about patient safety issues. Research on how nursing master students experience patient safety issues during their clinical placements is lacking and, consequently, educational programs may not be addressing patient safety issues as optimally as they could.

### Aim

The aim of this study was to explore nursing master students’ experiences and reflections on patient safety issues and adverse events during and after clinical practices.

The research questions were.

1. *To what extent do nursing master students observe, experience and discuss patient safety issues and adverse events during their clinical practice?*
2. *How do nursing master students reflect on patient safety issues and adverse events during and after clinical practice?*
3. *How do nursing master students experience using EMA to capture patient safety issues and adverse events?*

## Methods

### Design

The conceptual model of this study views learning as fundamentally anchored in social and physical contexts. Because of the situated nature of human thinking, a research approach which builds on the sampling of data in the clinical context, over time, was chosen (Reis et al., 2014). The study had a mixed-methods convergent design: Quantitative and qualitative data were collected simultaneously, analyzed separately and then merged [24, 25] Collecting different kinds of data over time has a potential of providing a fuller, deeper, wider and better understanding of the world [26].

### Setting and participants

The study was conducted at a Norwegian university college within the master programs in NA, CCN and ORN. In total, 109 master students were enrolled in the master programs: 31 NA students, 51 CCN students and 27 ORN students, respectively. The first author informed about the study in a lecture. Then, the students were invited to participate through their digital learning platform. Two e-mail reminders were sent in collaboration with the program leaders. An inclusion criterion was being a student of the NA, CCN or ORN master program. There were no exclusion criteria. The students who were interested in participating responded to the first author by e-mail. In total, 16 students participated in the EMA, and 10 of these students also participated in the interviews for the experiences of using EMA.

### Data collection

Both quantitative and qualitative data collection were performed using an EMA application (app) and a Notification Initiated System (NIS) which were downloaded to the students’ mobile phones. The data were stored on a safe platform anonymously [27]. As existing survey instruments that are suitable for EMA studies about patient safety and adverse events were not available, alternative options needed to be explored. Hence, the items had to be adapted and developed for this study and its clinical context. A balance needed to be found between gathering sufficient information and not overburdening students with an overly lengthy questionnaire [20]. The EMA statements followed suggestions by previous research regarding the type of items (assessment of statements using Likert scales followed by free text commenting), number of statements, length of data-collection, reminders and format [17, 23, 28]. To ensure the relevance of the questions, the ‘Checklist for optimally formulated ESM questions, as proposed by Eisele et al. [20], was followed.

A pilot study was conducted to assess the feasibility of the data-collection, the performance of the system, the phrasing of the questions, frequency of reminders and transferal of the data from the system afterwards. One master student from each specialty (all female) at another Norwegian university college participated. The pilot showed that responding to the queries in the app was considered easy, and that the questions were relevant and understandable. Based on the feedback, minor adjustments were made.

Quantitative data was collected through a survey of six statements in the EMA app repeated daily over a period of 14 days, which were responded to by using a Likert scale ranging from 1 to 5 (1 = totally disagree, to 5 = totally agree).

Qualitative data were collected through daily free-text reflections on the EMA app during clinical placements, as well as the semi-structured interviews thereafter. The six statement survey was followed by a free-text question allowing students to add reflections about their experiences. The EMA app served the dual purpose of collecting data about specific topics, but also to prompt students to comment on their own work experiences, observations and discussions about patient safety and near misses. Table 1 gives an overview of the EMA statements used.

**Table 1:** Daily statements assessed in the EMA app followed by a free-text question

1. I have observed an error or an event today that may pose a patient risk
2. I have discussed patient safety issues with others today (e.g. supervisors/colleagues/other students)
3. I have avoided an adverse event today because an error was discovered through the established procedures for patient safety (e.g. Safe Surgery Checklists and medication control)
4. I have experienced an error or a near miss concerning my work today
5. My work has been safe for the patient today
6. My work has threatened patient safety today
7. Free text: Do you have comments concerning your experiences today?

The data collection lasted 14 days during the students’ second or third clinical practice period, during one of three different periods offered, in June, August/September and November/December in 2022. A standard week of clinical placement consists of a minimum of 30 h of work, usually divided into four days of clinical practice and one day of self-study [12–14]. The number of days a week might be higher if the students choose to follow the supervisor’s rotation, resulting in different numbers of days of data collection/responses during the 14-day data collection periods. The participants received daily reminders through the app. To ensure that the daily reminders would not disturb the students in their clinical work, it was decided that the data collection would take place once a day, shortly after their shifts. The first reminder was given at 4 pm for students attending day shifts, and the next at 10.30 pm for students attending evening shifts. A follow-up reminder was sent after another hour. The students could respond later in the day, or if they wished to provide additional information, they could respond twice a day, but it was not possible to go back to previous days.

All the students participating in the EMA study were invited to participate in an interview. For pragmatic reasons, both focus groups and individual interviews were used as appropriate. An interview guide was developed based on the statements used in the EMA app, but also allowing the respondents to elaborate on the experiences and reflections of patient safety issues that they had reported, why the participants believed adverse events occurred, their learning about patient safety issues, and their experiences of using the EMA app as a method of data collection in clinical placements. This is presented in the Supplemental 1. Interviews were conducted by the first author (MVO), and since students were at three different campuses, both face-to-face and online meetings were offered. The face-to-face meetings were arranged in a meeting room at one of the hospitals where the participants had their clinical placements. The digital meetings were arranged as Zoom meetings so that participants could both see and hear each other. The interviews lasted between 19 and 60 min (median 51 min), and they were audio recorded. The interviews were transcribed verbatim by the first author shortly after the interviews.

### Analysis

Quantitative data were analyzed using descriptive statistics to quantify statement scores and sample characteristics. Specifically, means and standard deviations (SD) were calculated for statement scores, whereas counts and percentages (%) were calculated for sample characteristics (all categorical). We also made histograms to visualize the overall distribution of statements scores and further presented mean trend lines of statements scores from the first day of observation until seven consecutive days during the 14-day period. The time trends were estimated using linear mixed effects models with time as a linear independent variable and statements scores as the continuous dependent variable. Dependency in statement scores from the same individual over time was accounted for by specifying a random intercept for each individual in the regression models. The trend analyses also included measurements for individuals who reported more than once in a single day (five individuals). The P value for the trends was estimated by comparing models with and without the linear time variable by using the likelihood ratio test. Correlations between pairs of EMA statements were estimated using the method of repeated measure correlation (r) [29]. All statistical analyses and data visualization were performed using R Core Team R [30]. The Statistical Package for the Social Sciences (IBM SPSS Statistics, Version 29) was used when making an overview of the free-text responses, and for describing demographics of the students participating in the interviews afterwards.

Qualitative data, the free-text responses and the interview data were analyzed using Braun and Clark’s recommendations for thematic analysis [31, 32]. The free-text responses and the transcribed interviews were analyzed separately following these four steps: 1) Familiarization through the first author reading free-texts/transcripts in their entirety to get an overview of the contents (MVO). 2) The texts were then re-read to find patterns in the respondents’ experiences and reflections, including the coding of keywords and terms of interest, guided by the research questions, by the first authors and co-authors (MVO, KK and ACLL). 3) Then, the codes were explored, searching for themes across the data (MVO, KK and ACLL). Finally, the transcripts were read repeatedly while revising the codes and the themes (MVO). The codes and themes were discussed by three of the authors (MVO, KK and ACLL) until consensus was reached. Table 2 shows a part of this process.

**Table 2:** The process of analyzing the free-text responses and interviews

Free text response	Codes	Theme
*I was mostly observing in my clinical placement today. When a child was given anesthesia, his oxygen saturation dropped to 79%. At first, we (NA, NA student and anesthetist) didn’t understand the reason, but then we discovered that room air, not oxygen, was administrated to the patient. They had an MR anesthesia machine, which is unfamiliar to many* (4_NA student, female).	Observing Child and anesthesia Low oxygen saturation Could not find the reason Registration of air and oxygen Anesthesia machine Lack of competence	Experiences of adverse events or near misses

**Interview**	Codes	Theme
*I have discussed a lot with the supervisors about what patient safety is and what it is not. If I hadn’t used the EMA app, I don’t think I had thought that much about…actually, regarding how things are done… *(3-CCN student, male).	Discussed patient safety with people in the team The EMA app drew attention to how things were done.	Increased awareness

## Results

### Participants’ demographics

Table 3 shows the demographics of the participants: specialty, gender, age and number of years of experience as a registered nurse before entering the master program, were collected the first time the students used the app. Most NA students participated in the study and less males. More than half of the participants were less than 30 years and had more than two years of experience as nurses before starting at the master nursing program.

**Table 3:** Participant demographics

	**EMA**	**Interviews**
	n (%)	n (%)
Participants	16	10
Specialty		
Nurse anesthesia	9 (56%)	5 (50%)
Critical Care Nursing	5 (31%)	4 (40%)
Operating room nursing	2 (13%)	1 (10%)
Gender		
Male	5 (31%)	3 (30%)
Female	11 (69%)	7 (70%)
Age		
Younger than 30 years	9 (56%)	7 (70%)
30–39 years	6 (38%)	3 (30%)
40–49 years	1 (6%)	–
Experience as a registered nurse		
Less than 2 years	1 (6%)	1 (10%)
2–4 years	8 (50%)	6 (60%)
5–9 years	5 (31%)	3 (30%)
More than 10 years	2 (13%)	–

*Abbreviation*: *EMA* Ecological Momentary Assesment

### Quantitative results from the ecological momentary assessment

In total, 105 EMA responses were included in the analysis. The students submitted between four and nine responses each during the 14-day period, and the results are presented in Table 4.

**Table 4:** Mean responses to the EMA statements

**Statement**	**1** **Observed** Mean (SD)	**2** **Discussed** Mean (SD)	**3** **Avoided** Mean (SD)	**4** **Experienced** Mean (SD)	**5** **Safe work** Mean (SD)	**6** **Threatened** Mean (SD)
Overall	2.5 (1.6)	3.9 (1.4)	2.5 (1.6)	2.0 (1.4)	4.6 (0.9)	1.4 (0.8)

*Abbreviation*: *SD* Standard deviation

Statements: 1. Observed an error; 2. Discussed patient safety issues; 3. Avoided an adverse event; 4. Experienced an error or near-miss; 5. Safe work for the patient, and 6. Work has threatened patient safety. Likert scales (1-5) were used ranging from 1= Totally disagree to 5= Totally agree

Overall, the students mostly disagreed to or were neutral with the statement that they had observed an error or an event that may pose a patient safety risk today (Statement 1. Observed: mean 2.5). They, however, did mostly agree about having discussed patient safety issues during the day with supervisors/colleagues/other students, (Statement 2. Discussed: mean 3.9). They mostly disagreed to or were neutral about, having avoided adverse events because errors were discovered through established procedures for patient safety (Statement 3. Avoided: mean 2.5). They also mostly disagreed about having experienced errors or near-misses concerning their work during the day (Statement 4. Experienced: mean 2.0). Finally, they totally or mostly agreed with the statement that their work had been safe for the patient during the day (Statement 5. Safe work: mean 4.6) and mostly or totally disagreed with the statement that their work had threatened patient safety during the day (Statement 6, Threatened: mean 1.4). The results showed that there were no differences between the age groups, gender, specialty and experiences as nurses concerning the six daily EMA statements. The details of these results are presented as mean responses to the EMA statements and covariation with demographics in Supplemental 2.

**Fig. 1:**
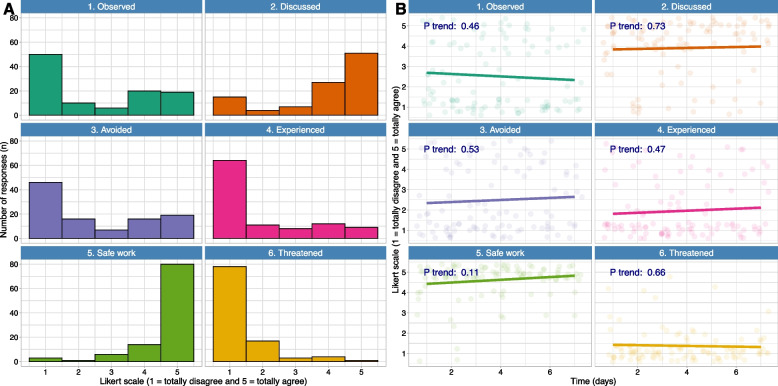
Results from the daily responses visualized as overall distributions on the left (**A**) and mean time trends on the right (**B**)*.* Dots in panel B show the individual responses at each time point, slightly jittered to avoid overplotting of data points

The distributions of the responses showed that there was variation among the answers (Fig. 1). Diverging from the mean responses and the trend lines, some participants observed errors or events that could have posed patient safety risks (Statement 1. Observed). Similarly, some participants reported having avoided adverse events by using established procedures for patient safety such as checklists or double medication control (Statement 3. Avoided) or they confirmed that they had experienced adverse events or near-misses concerning their work (Statement 4. Experienced). Fig. 1 (B) also showed trend lines for each statement, but no strong changes in the trends were observed for the statements during the observations period (*p* values > 0.10).

The strength of the correlation between pairs of EMA statements were estimated using the method of repeated measure correlation, see Table 5.

**Table 5:** Repeated measure correlation (r) between pairs of EMA statements

	1.Observed	2. Discussed	3. Avoided	4. Experienced	5.Safe work	6. Threatened
1.Observed	1					
2. Discussed	0.430	1				
3. Avoided	0.385	0.173	1			
4. Experienced	0.624	0.414	0.308	1		
5. Safe work	−0.180	−0.208	0.073	−0.271	1	
6. Threatened	0.318	0.296	0.012	0.355	−0.275	1

Statements: 1. Observed an error; 2. Discussed patient safety issues; 3. Avoided an adverse event; 4. Experienced an error or near-miss; 5. Safe work for the patient, and 6. Work has threatened patient safety

The strongest positive correlation (r = 0.624) was between experiencing an error or near miss (Statement 4. Experienced) and observing an error or an event that may pose a patient risk (Statement 1. Observed). The strongest inverse correlation (r = −0.275) was between work having threatened patient safety (Statement 6. Threatened) and experiencing work to be safe for the patient (Statement 5. Safe work). In contrast, there were no correlations between safe work for the patient (r = 0.073) (Statement 5. Safe Work) and having avoided adverse events today (Statement 3. Avoided), or between threatened patient safety (r = 0.012) (Statement 6. Threatened) and having avoided adverse events today (Statement 3. Avoided).

### Qualitative results

#### Reflections during clinical practice

Eleven of the students provided free-text comments during the two-week clinical placements. Comments were given by all nursing specialties as well as by males and females. A total of 44 comments were provided, expressing both positive experiences and near misses. Three themes were identified: 1) *Adverse events or near misses*, 2) *Communication and interactions in the team* and 3) *Using security procedures*. The findings are illustrated with quotes from the students.

*Adverse events or near misses* related to the participants’ experiences of specific adverse events or near misses during their clinical placements. These events included erroneous medication administration or double control, improper subcutaneous administration of intravenous infusion, incorrect use of medical equipment, loss of surgical equipment, clogged urinary catheters, loss of human resources and suboptimal communication in the teams. The issues were perceived as near misses. One of the students commented:

*Problems with the equipment were discovered during the surgery. The patient had to be anesthetized longer than necessary, while new equipment was organized. The problems had been reported earlier but had not been fixed. Reported in a proper way today* (12_ORN student, female).

Another student wrote:

*We used a syringe pump for administering blood pressure medication and discovered that the wrong syringe had been registered (both the brand and the size). If this had not been discovered, the infusion could have ended without giving a “soon empty syringe” alarm. This was discovered by using a checklist* (7_CCN student, female).

*Communication and interactions in the team* related to discussions with other team members. The supervisor was the person most commonly discussed with, but also conversations with both the anesthesiologists and the surgeons were described. The main focus concerned risk assessments in different situations, e.g., emergency procedures during the transportation of a critical care patient, establishing a Plan A and a Plan B during anesthesia, risk prevention concerning bleeding and danger of anaphylaxis, pressure ulcer prevention and sterility in the OR. One of the students commented:

*We discussed emergency preparedness concerning the possibility of bleeding and agreed on several measures, including ordering blood in advance. In addition, since the patient was to receive contrast that could potentially cause anaphylaxis, we ensured that fluid with a pressure cuff and emergency medications were readily available* (15_NA student, female).

Different factors related to communication in the team were also mentioned as safety threats. For instance, one of the students commented:

*I observed a lack of communication during the procedure, which lead to a misunderstanding and an adverse event* (8_CCN student, female).

*Using established security procedures* showed how the students observed the use of procedures or used these themselves in clinical practice, such as the SSC. The students expressed that procedures were of great use, and they gave examples of how using these helped them avoid adverse events. One of the students made the following comment:

*Our operation room program was delayed today because of a missing pre-transfusion screening test. The situation was discovered using the SSC before the patient entered the operation room* (1_NA student, female).

They also experienced that some colleagues lacked interest in safety procedures such as the SSC, and that it was necessary to make own decisions regarding safety. One of the students gave this example:

*Some surgeons don’t see the point of taking part in the SSC, and they just start before you have done the check of the list…at that point, I raised my voice and started the check, and they joined in, but it was obvious that it was of little interest to them* (11_NA student, female).

Despite the overall focus being on patient safety and adverse events, some students also exemplified situations which were good and positive experiences for them. One of the students reported:

*There have been no adverse events or near misses today. We have reflected on patient safety before each surgical procedure, and we prepared both Plan A and a Plan B, when necessary* (4_NA student, female).

Another student gave this example:

*We have expressed good “time outs” both before and after the surgical procedures today using the checklist. The surgeon included everyone in the team and noted when everyone was ready to take part in the session* (1_NA student, female).

Table 6 shows examples of how the quantitative EMA assessment related to students’ reflections during the clinical practice. When the students had made observations of errors and events posing patient risks (high ratings of Statement 1), they often commented on these in free- text, e.g., faulty, malfunctioning or mishandled equipment (syringes, central venous lines, infusion), communication errors, or lack of interest in carrying out safety procedures. When they reported that they had not made such observations (low rating of Statement 1), they would also comment in free text, but then instead about how work had been calm and risk-free. They would also comment on how patient safety issues had been managed successfully (e.g., by using checklists, preparing, and by carrying out good "time outs"). A similar pattern was seen when the students assessed whether they had discussed with others or experienced patient safety issues and errors the same day (Statements 2 and 4). When they had observed patient safety issues the students reported also having discussed patient safety issues, but the opposite was not necessarily always the case. When they had experienced an error or a near miss, they also reported having observed and discussed it. However, not all errors and patient safety issues that were observed or discussed were reported as events that were experienced in their work.

**Table 6:** Responses to the EMA statements and free text comments

Participant	Responses of the EMA statements							Free Text Comments given
	1.Observed	2. Discussed	3. Avoided	4. Experienced	5.Safe work	6. Threatened		
								Adverse events or near misses
12_ORN student, female	4	5	2	2	3		2	*Problems with the equipment were discovered during the surgery. The patient had to be anesthetized longer than necessary, while new equipment was organized. The problems had been reported earlier but had not been fixed. Reported in a proper way today*
7_CCNstudent, female	5	5	5	2	5		1	*We used a syringe pump for administering blood pressure medication and discovered that the wrong syringe had been registered (both the brand and the size). If this had not been discovered, the infusion could have ended without giving a “soon empty syringe” alarm. This was discovered by using a checklist*
03_CCN student Male	1	1	1	1	5		1	*The shift was quiet, with no situations that posed any risk to the patient*
03_CCN student Male	5	5	1	5	3		1	*The patient had received an infusion due to hypotension; however, the infusion had infiltrated subcutaneously, resulting in leakage into the surrounding tissue. Fortunately, only saline (NaCl) was involved, and no medications such as potassium were administered. The infusion was discontinued, and the peripheral venous catheter was removed. An arterial line was also present in the same arm, which was automatically removed due to the swelling. I consulted with the doctor, and aside from applying a compression bandage, no additional precautions were deemed necessary*
03_CCN student Male	5	5	5	5	5		1	*Air was detected in the central venous line*
								Communication and interactions in the team
15_NA student, female	1	5	4	1	5		1	*We discussed emergency preparedness concerning the possibility of bleeding and agreed on several measures, including ordering blood in advance. Additionally, since the patient was to receive contrast that could potentially cause anaphylaxis, we ensured that fluid with a pressure cuff and emergency medications were readily available*
8_CCN student, female	5	5	2	2	5		2	*I observed a lack of communication during the procedure, which led to a misunderstanding and an adverse event*
								Using established security procedures
1_NA student, female	1	5	5	1	5		1	*Our operation room program was delayed today because of a missing pre-transfusion screening test. The situation was discovered using the SSC before the patient entered the operation room*
11_NA student, female	5	5	2	1	5		1	*Some surgeons don’t see the point of taking part in the SSC, and they just start before you have done the check of the list…at that point, I raised my voice and started the check, and they joined in, but it was obvious that it was of little interest to them*
4_NA student, female	1	5	1	1	5		1	*There have been no adverse events or near misses today. We have reflected on patient safety before each surgical procedure, and we prepared both Plan A and Plan B, when necessary*
1_NA student, female	1	5	1	1	5		1	*We have expressed good “time outs’” both before and after the surgical procedures today using the checklist. The surgeon included everyone in the team and noted when everyone was ready to take part in the session*
03_CCN student Male	2	5	3	3	3		3	*Today, I attended the OR as a CCN student. I served as an observer and did not engage in any activities that could pose a risk to the patient. Safe surgery protocols were discussed in the OR, and the Surgical Safety Checklist (SSC) was utilized*

*Abbreviations*: *EMA* Ecological Momentary Assessment, *NA* Nurse Anesthesia, *CCN* Critical Care Nurse, *ORN* Operation Theatre Nurse

Statements: 1. Observed an error; 2. Discussed patient safety issues; 3. Avoided an adverse event; 4. Experienced an error or near-miss; 5. Safe work for the patient, and 6. Work has threatened patient safety. Likert scales (1–5) were used ranging from 1 = Totally disagree to 5 = Totally agree

When the students reported that they had avoided adverse events because they had discovered errors (high ratings of statement 3), they would typically suggest that they had had help of checklists such as the SSC if any explanation was provided to how they managed to avoid the event. Patterns could not be detected when ratings were low, but these cases would cover all other cases which could logically vary highly in character (both risky and not risky events).

Students consistently rated that their patient had been safe and that it had not posed a threat. They did not provide low scores about safety, but in the cases when they only gave an average rating of 3 on the statements relating to patient safety, they referred to trouble with the equipment or commented that they only had been observers.

### Reflections after clinical practice

One overall theme relating to the research questions was identified through the analysis of the interviews, namely *Increased awareness*. All the students reported that the daily reporting through the EMA app had made them focus on patient safety in a way they had not done before. After their shifts, they reflected on what they had experienced, and such reflection after action was seen as valuable, regardless of whether extraordinary events took place or not. The students agreed that continuously raising awareness about patient safety was effective. One of the students expressed it like this:

*In my opinion, the EMA app was of great use. Even if there were no adverse events at this time, you were more aware of them. And… what is also good is that you pay more attention to what others do well. The daily registration in the app was a safety procedure in itself because it drew attention to the patient safety issues* (8_CCN student, female).

Summing up, their experiences and reflections of the day as well as what they had seen and done were considered a novel activity contributing to learning in the clinical placements. One of the students said:

*I definitely mostly focused on adverse events. You felt it in the beginning, when starting the app, and you were thinking about the day that maybe there are more than just adverse events… You start reflecting…then you think about the whole day and what you actually had done. It was very exciting* (2_NA student, male).

Students seemed surprised when they noticed that there were not just negative things to reflect on concerning patient safety – mostly things were done correctly. Still, they expressed that good work, and positive experiences received less emphasis, but they encouraged themselves when they felt that things went well. One of the students said:

*You go through the day in a different way. Maybe in a different way than you usually do. You sort of think about what you have done, so…sometimes it’s a bit difficult too… I think my thoughts were like…what is ****not**** patient safe in a way, but most of what we do...it is procedures. I don’t feel like we do a lot of things that are not...yes like...okay…* (8_CCN student, female).

## Discussion

Our study indicated that few adverse events were observed in the clinical practice periods. However, the students’ reflections during clinical practice expanded this impression by presenting examples of such events. After the clinical training periods, students appreciated the increased awareness that use of the EMA app led to regarding their learning and patient safety issues.

During the clinical practice periods, the students provided reports about incidents relating to erroneous medication, improper fluid administration, incorrect use and loss of equipment as well as suboptimal teamwork and communication. In the light of recent reports indicating that about one in ten patients are harmed in healthcare, it may not be surprising that students observe and experience safety errors in their clinical placements [1]. This is also in line with Hwang et al. [33], who found that one third of participating students experienced errors during their clinical placements. Espin et al. [34], reported that nursing students had witnessed or reported 3.8 incidents per 1,000 days of training, mainly related to medication administration. About one third of fourth-year nursing students described having experienced medication errors or near misses. Schwendimann et al. [35], found that most reported adverse events were operative/surgical related, medication errors, health care associated infections and allergic reactions, and these findings align with the events reported in the present study. The WHO [36], defines three kinds of incidents: near misses (the incidents do not reach the patient), no harm incidents (the incidents reach the patient but do not cause harm) and harmful incidents (the incidents result in harm for the patient). In the current study, the incidents were “near misses” or “no harm incidents” according to these terms.

In the present study clear communication and interactions in the teams was an important patient safety theme brought up by the students. The supervisors were highlighted as the most common discussion partners. Bump et al. [37] found that trainees rated teamwork across and within units, and supervisors or managers promoting patient safety as aspects that contributed to patient safety. Espin et al. [34], also found that supervision was an important requirement for safer clinical practice. In a study by Glarcher et al. [38], advanced practice nurses expressed that “being a role model” to other team members, especially junior staff, and to “act as a change agent or leader” especially contributed to patient safety in clinical practice. They also underlined the importance of cooperating with colleagues about safe care in order to ensure that all were in line and had the same plan for the patients.

The students also commented that there were different ways of carrying out the SSC and medication control. They explored different ways to manage procedures concerning these and gave positive examples of preventing near misses in some of the cases, as well as negative examples of when procedures were ignored in others. Armstrong et al. [39] explored the providers and patients’ outcomes using checklists and found that the impact of the SSC would be better understood if the quality of how the checklist was used would be explored. *How* checklists are completed matters more than *if* they are completed. Haugen et al. [40], pointed out the importance of the WHO’s SSC to improve teamwork, communication and consistency of care in order to ensure that all individuals involved in the patient treatment adhere to the same guidelines, protocols and procedures to achieve positive outcomes and prevent errors or unnecessary complications. Hence, the students’ experiences of not performing the SSC adequately may be seen as near misses.

Using the EMA methodology to capture learning about patient safety issues in a clinical context is a rather novel research approach. The EMA approach provided numerous detailed accounts about events relating to patient safety and near misses that would most likely have been difficult to capture using retrospective methods. Previous research has found the EMA approach to be suitable for collecting data about students’ experiences in clinical learning environments [17, 23], and as a method in a curriculum revision project [41]. Here, the EMA method was promoted as an important contribution to education by allowing programs to demonstrate continuous improvement in their educational processes.

Of interest in the present study was how students connected their involvement in the EMA to both becoming more aware about patient safety and adverse events, but also about the many things that went well during clinical practice. The participants indicated various ratings (high/low) on the given EMA statements, concerning different situations and patient safety issues presented (Table 6). Different kinds of problems, risks, and challenges were brought up in their free text comments. The participants rated in unison that their work had been safe (M = 4.6) and had not threatened patient safety (M = 1.4). Even if they reported having avoided an adverse event, they considered their work as being safe and not having threatened the patient’s safety (cf. Table 6). One interpretation is that avoiding potential adverse events by using patient safety procedures such as checklists is considered a normal routine, rather than an extreme exception. Another interpretation is that they considered *their* own work as safe, but that they considered the work of others as unsafe or patient threatening. Interestingly, while the questions did not specifically prompt for it, their comments also showed that they reflected on work and collaboration that functioned well, e.g., when they experienced work as calm, when they prepared their work to avoid safety threats, when they carried out "timeouts", or when they used checklists. This observation thus links to the distinction between Safety I and Safety II approaches and having a focus on “learning from what goes wrong” vs. “learning from what goes well” [42]. A Safety I approach in line with WHO’s patient safety curriculum, highlights experiences and reflections about adverse events and near misses, but this approach has been put under question for not sufficiently considering recent theory about Resilient Health Care and there may indeed be value in also systematically capturing students’ positive experiences in future research [43, 44].

Students in the present study also reported that using the EMA app inspired them to ask about and discuss patient safety themes, mainly with their supervisor, but also with others in their team. The app was experienced as flexible, once they had become familiar with it, and they experienced it as easy to use. The daily reports were completed in about five minutes. They reflected individually while writing notes, but comments and examples could also be shared and discussed in meetings with clinical instructors and other students. For translating principles of resilience into practice Haraldseid-Driftland et al. [45], explored the key learning principles for tools to help this translation. These learning principles are good recommendations and may also be transferable to the use of the EMA application as well as the evaluation given. The app could thus be used as a method to encourage and support experiences and reflections on patient safety from both the Safety I and Safety II perspective and as a good starting point for group reflection with both supervisors and clinical instructors [42].

The students in this study have a theoretical introduction to the theme of patient safety before clinical placement as well as the follow-up by clinical placement afterwards. Hwang et al. [33], addressed patient safety in education by offering final year medical and nursing students a 1-day patient safety course which raised their competence about patient safety. Elguea et al. [46], used the result of a survey to define the changes needed to be implemented in the patient safety learning, as well as to point out that perceptions of safety also affected the students’ attitude to safety in clinical practice. In Norway, establishing patient safety as a compulsory part of the national guidelines of the master program of NA, CCN and ORN students is an even stronger guide in the work of patient safety [12–14]. Still, the content of this curriculum will be crucial for the diversity of knowledge to be conveyed, and the learning methods used need to be focused on.

### Strengths and limitations

A strength of this study is the mixed method approach, enabling the convergence of different views and input from the students. The quantitative data gave an overview of patient safety issues and experiences of adverse events during the students’ clinical placements, while the qualitative data gave a more nuanced picture of these experiences. A larger sample size and a better representation of especially ORN students, as well as a more balanced number of genders, could have enabled the collection of observations and views that may have been missed in this study. Still, the imbalance between women and men reflected here also mirrors a situation that is common in the Norwegian healthcare system in general, where there is usually a considerably larger proportion of women. Using EMA allowed data collection in close connection to clinical experiences, reducing the bias of time, and possibilities of overlooking details. Collecting data over time (14 days), also gave an increased number of responses, which could compensate for the low number of participants. Having the same students participate in all parts of the study strengthens the results, as this has allowed integrating opinions and observation collected in different ways and at different points of time. Also, the clinical settings with new patients, situations and places every day might give daily new input.

A limitation of this study is the number of students participating compared to the total number of students at the master program. A larger number of participants could potentially have contributed with more observations and insights. Thus, van Berkel et al. [22] pointed in their historical overview of EMA on the number of participants, which they indicated to a median number of 19, not far from this study. Despite the limited number of participants, the combination of methods provided extensive data contributing to the validity and trustworthiness of our results.

## Conclusions

Using EMA for reporting about adverse events and near misses in clinical placements initially indicated a low occurrence of such events. However, the EMA reports captured a number of such events showing the continued need to further address patient safety issues during the training of nursing master students. The EMA data could potentially provide input about authentic incidents with high relevance to being addressed in healthcare education. Moreover, involvement in data collection using the EMA method increased the students’ awareness of both adverse events and near-misses as well as work that was performed well. This method should be further tested and evaluated as a tool for reflection on patient safety in clinical placements during the education of master nursing students and other healthcare students.

## Supplementary Information

Supplementary Material 1.

Supplementary Material 2.

## Data Availability

All data generated or analyzed during this study are included in this published article and its supplementary information files.

## Abbreviations

EMA: Ecological Momentary Assessment
CCN: Critical Care Nurse / Critical Care Nursing
NA: Nurse Anesthesia / Nurse anesthetist
NIS: Notification Initiated System
ORN: Operation Room Nurse / Operation Room Nursing
SD: Standard Deviation
SSC: Safe Surgery Checklist
WHO: World Health Organization

## References

[1] WHO. Patient Safety https://www.who.int/news-room/fact-sheets/detail/patient-safety: World Health Organization,WHO; 2023 .Available at: Patient safety.

[2] Sauro KM, Machan M, Whalen-Browne L, Owen V, Wu G, Stelfox HT. Evolving factors in hospital safety: a systematic review and meta-analysis of hospital adverse events. J Patient Saf. 2021;17(8):e1285–95. 34469915 10.1097/PTS.0000000000000889

[3] Wæhle HV, Haugen AS, Wiig S, Søfteland E, Sevdalis N, Harthug S. How does the WHO surgical safety checklist fit with existing perioperative risk management strategies? An ethnographic study across surgical specialties. BMC Health Serv Res. 2020;20(1):111. 10.1186/s12913-020-4965-5. 32050960 10.1186/s12913-020-4965-5 PMC7017532

[4] Holmes T, Vifladt A, Ballangrud R. A qualitative study of how inter-professional teamwork influences perioperative nursing. Nurs Open. 2020;7(2):571–80. 10.1002/nop2.422. 32089854 10.1002/nop2.422 PMC7024613

[5] Reine E, Aase K, Raeder J, Thorud A, Aarsnes RM, Rustoen T. Exploring postoperative handover quality in relation to patient condition: a mixed methods study. J Clin Nurs. 2021;30(7–8):1046–59. 10.1111/jocn.15650. 33434381 10.1111/jocn.15650

[6] Leonardsen A-CL, Moen EK, Karlsøen G, Hovland T. A quantitative study on personnel’s experiences with patient handovers between the operating room and the postoperative anesthesia care unit before and after the implementation of a structured communication tool. Nursing reports (Pavia, Italy). 2019;9(1). 10.4081/nursrep.2019.8041

[7] Amaniyan S, Faldaas BO, Logan PA, Vaismoradi M. Learning from patient safety incidents in the emergency department: a systematic review. J Emerg Med. 2020;58(2):234–44. 10.1016/j.jemermed.2019.11.015. 31843322 10.1016/j.jemermed.2019.11.015

[8] Huston CL, Phillips B, Jeffries P, Todero C, Rich J, Knecht P, et al. The academic-practice gap: strategies for an enduring problem. Nurs Forum. 2018;53(1):27–34. 10.1111/nuf.12216. 28815609 10.1111/nuf.12216

[9] Karlgren K, Lakkala M, Toom A, Ilomäki L, Lahti-Nuuttila P, Muukkonen H. Assessing the learning of knowledge work competence in higher education - cross-cultural translation and adaptation of the collaborative knowledge practices questionnaire. Res Pap Educ. 2020;35(1):8–22. 10.1080/02671522.2019.1677752.

[10] Weeks KW, Coben D, O’Neill D, Jones A, Weeks A, Brown M, et al. Developing and integrating nursing competence through authentic technology-enhanced clinical simulation education: pedagogies for reconceptualising the theory-practice gap. Nurse Educ Pract. 2019;37:29–38. 10.1016/j.nepr.2019.04.010. 31060016 10.1016/j.nepr.2019.04.010

[11] Tella S, Smith N-J, Partanen P, Turunen H. Work placements as learning environments for patient safety: Finnish and British preregistration nursing students’ important learning events. J Vocat Educ Train. 2016;68(1):51–69. 10.1080/13636820.2015.1104715.

[12] Forskrift om nasjonal retningslinje for anestesisykepleierutdanning. Availaible at: Forskrift om nasjonal retningslinje for anestesisykepleierutdanning - Lovdata (The Norwegian legal database). 2021.

[13] Forskrift om nasjonal retningslinje for intensivsykepleierutdanning. Availaible at: Forskrift om nasjonal retningslinje for intensivsykepleierutdanning - Lovdata(The Norwegian legal database). 2021.

[14] Forskrift om nasjonal retningslinje for operasjonssykepleierutdanning. Availaible at: Forskrift om nasjonal retningslinje for operasjonssykepleierutdanning - Lovdata (The Norwegian legal database). 2021.

[15] Reis H, Gable S, Maniaci M. Methods for Studying Everyday Experience in Its Natural Context. Cambridge University Press; 2014. p. 373–403. 10.1017/CBO9780511996481.019.

[16] Barrett LF, Barrett DJ. An introduction to computerized experience sampling in psychology. Soc Sci Comput Rev. 2001;19(2):175–85. 10.1177/089443930101900204.

[17] Lachmann H, Ponzer S, Johansson U-B, Karlgren K. Introducing and adapting a novel method for investigating learning experiences in clinical learning environments. Inform Health Soc Care. 2012;37(3):125–40. 10.3109/13561820.2014.907777. 22713123 10.3109/17538157.2012.678449

[18] Kosonen K, et al. Research on knowledge practices with the Contextual Activity Sampling System. Proceedings of the 9th international conference on Computer supported collaborative learning-CSCL'09. 2009. Avaliable at: https://www.academia.edu/350264/Muukkonen_H_Inkinen_M_Kosonen_K_Hakkarainen_K_Karlgren_K_Lachmann_H_and_Vesikivi_P_2009_Research_on_knowledge_practices_with_the_Contextual_Activity_Sampling_System?sm=b&rhid=36412833332.

[19] Christensen TC, Barrett LF, Bliss-Moreau E, Lebo K, Kaschub C. A practical guide to experience-sampling procedures. J Happiness Studies. 2003;4(1):53–78. ISSN: 1573–7780.

[20] Eisele G, et al. Questionnaire design and evaluation. The open handbook of experience sampling methodology: A step-by-step guide to designing, conducting, and analyzing ESM studies. Myin-Germeys I and Kuppens P: 2021. 71–90.

[21] Liao Y, Skelton K, Dunton G, Bruening M. A systematic review of methods and procedures used in ecological momentary assessments of diet and physical activity research in youth: an adapted STROBE checklist for reporting EMA studies (CREMAS). J Med Internet Res. 2016;18(6):e151. 10.2196/jmir.4954. 27328833 10.2196/jmir.4954 PMC4933800

[22] Van Berkel N, Ferreira D, Kostakos V. The experience sampling method on mobile devices. ACM Computing Surveys (CSUR). 2017;50(6):1–40. 10.1145/3123988.

[23] Karlgren K, Andersson Franko M, Kilström D. Experiencing one thing and saying another’–ecological momentary assessment (EMA) of nursing students’ competence and challenge during clinical placements compared with retrospective interviews. PLoS One. 2024;19(5):e0302866. 10.1371/journal.pone.0302866. 38776304 10.1371/journal.pone.0302866 PMC11111015

[24] Fetters MD, Curry LA, Creswell JW. Achieving integration in mixed methods designs—principles and practices. Health Serv Res. 2013;48(6pt2):2134–56. 10.1111/1475-6773.12117. 24279835 10.1111/1475-6773.12117 PMC4097839

[25] Polit DF, Beck CT. Nursing research: Generating and assessing evidence for nursing practice. 11th ed. Philadelphia: Lippincott Williams & Wilkins; 2021. ISBN: 9781975154141,1975154142,9781975110642.

[26] Johnson B, Christensen LB. Educational research : quantitative, qualitative, and mixed approaches. Seventh edition.; International student edition. ed. Los Angeles: SAGE; 2020. ISBN: 9781544372174.

[27] Life Data. LifeData: 2021 Availiable at : Features - LifeData Experience Sampling App for Research.

[28] Mortensen M, Naustdal KI, Uibu E, Mägi L, Kangasniemi M, Põlluste K, et al. Instruments for measuring patient safety competencies in nursing: a scoping review. BMJ Open Qual. 2022;11(2):e001751. 10.1136/bmjoq-2021-001751. 10.1136/bmjoq-2021-001751 PMC8981364 35379672

[29] Bakdash JZ, Marusich LR. Repeated measures correlation. Front Psychol. 2017;8:456. 10.3389/fpsyg.2017.00456. 28439244 10.3389/fpsyg.2017.00456 PMC5383908

[30] R Core Team R: A language and environment for statistical computing. R Foundation for Statistical Computing, Vienna, Austria https://www.r-project.org/2024 [Available from: https://www.r-project.org/.

[31] Braun V, Clarke V. Toward good practice in thematic analysis: avoiding common problems and be(com)ing aknowingresearcher. Int J Transgender Health. 2023;24(1):1–6. 10.1080/26895269.2022.2129597. 10.1080/26895269.2022.2129597 PMC9879167 36713144

[32] Braun V, Clarke V, Braun V. Thematic analysis : a practical guide. Los Angeles, California: SAGE; 2022.

[33] Hwang J-I, Yoon T-Y, Jin H-J, Park Y, Park J-Y, Lee B-J. Patient safety competence for final-year health professional students: perceptions of effectiveness of an interprofessional education course. J Interprof Care. 2016;30(6):732–8. 10.1080/13561820.2016.1218446. 27705029 10.1080/13561820.2016.1218446

[34] Espin S, Sears N, Indar A, Duhn L, LeGrow K, Thapa B. Nursing students’ experiences of patient safety incidents and reporting: a scoping review. J Nurs Educ Pract. 2019;10(4):26. 10.5430/jnep.v10n4p26.

[35] Schwendimann R, Blatter C, Dhaini S, Simon M, Ausserhofer D. The occurrence, types, consequences and preventability of in-hospital adverse events–a scoping review. BMC Health Serv Res. 2018;18(1):1–13. 10.1186/s12913-018-3335-z. 29973258 10.1186/s12913-018-3335-z PMC6032777

[36] WHO. Patient safety incident reporting and learning systems; 2020. Report: Availiable at: Patient safety incident reporting and learning systems: technical report and guidance.

[37] Bump GM, Coots N, Liberi CA, Minnier TE, Phrampus PE, Gosman G, et al. Comparing trainee and staff perceptions of patient safety culture. Acad Med. 2017;92(1):116–22. 10.1097/ACM.0000000000001255. 27276009 10.1097/ACM.0000000000001255

[38] Glarcher M, Rihari-Thomas J, Duffield C, Tuqiri K, Hackett K, Ferguson C. Advanced practice nurses’ experiences of patient safety: a focus group study. Contemp Nurse. 2024. 10.1080/10376178.2024.2363911. 10.1080/10376178.2024.2363911 38861587

[39] Armstrong BA, Dutescu IA, Nemoy L, Bhavsar E, Carter DN, Ng K-D, et al. Effect of the surgical safety checklist on provider and patient outcomes: a systematic review. BMJ Qual Saf. 2022;31(6):463–78. 10.1136/bmjqs-2021-014361 35393355

[40] Haugen AS, Søfteland E, Sevdalis N, Eide GE, Nortvedt MW, Vincent C, et al. Impact of the Norwegian National Patient Safety Program on implementation of the WHO Surgical Safety Checklist and on perioperative safety culture. BMJ Open Qual. 2020. 10.1136/bmjoq-2020-000966. 10.1136/bmjoq-2020-000966 PMC7394019 32737022

[41] Willett L, Houston TK, Heudebert GR, Estrada C. Use of ecological momentary assessment to determine which structural factors impact perceived teaching quality of attending rounds. J Grad Med Educ. 2012;4(3):322–8. 10.4300/JGME-D-11-00265.1. 23997876 10.4300/JGME-D-11-00265.1 PMC3444185

[42] Hollnagel E, Wears RL, Braithwaite J. From Safety-I to Safety-II: a white paper. The resilient health care net: published simultaneously by the University of Southern Denmark, University of Florida, USA, and Macquarie University, Australia. 2015.At: https://resilienthealthcare.net/wp-content/uploads/2018/05/WhitePaperFinal.pdf.

[43] Sujan MA, Furniss D, Anderson J, Braithwaite J, Hollnagel E. Resilient health care as the basis for teaching patient safety – a Safety-II critique of the World Health Organisation patient safety curriculum. Saf Sci. 2019;118:15–21. 10.1016/j.ssci.2019.04.046.

[44] WHO. Patient safety curriculum guide. Multi-professional edition. World Health Organization; 2011. Report No.: 8555268508. ISBN 978 92 4 150195 8. Availiable at: Patient safety curriculum guide: multi-professional edition.

[45] Haraldseid-Driftland C, Lyng HB, Guise V, Waehle HV, Schibevaag L, Ree E, et al. Learning does not just happen: establishing learning principles for tools to translate resilience into practice, based on a participatory approach. BMC Health Serv Res. 2023;23(1):646. 10.1186/s12913-023-09653-8. 37328864 10.1186/s12913-023-09653-8 PMC10276446

[46] de Elguea JO, Orkaizagirre-Gómara A, De Miguel MS, Urcola-Pardo F, Germán-Bes C, Lizaso-Elgarresta I. Adapting and validating the Hospital Survey on Patient Safety Culture (HSOPS) for nursing students (HSOPS-NS): a new measure of Patient Safety Climate. Nurse Educ Today. 2019;75:95–103. 10.1016/j.nedt.2019.01.008. 30738365 10.1016/j.nedt.2019.01.008

[47] WMA. World Medical Accociation; Declaration of Helsinki: Ethical principles for medical research involving human subjects Available from: https://www.wma.net/what-we-do/medical-ethics/declaration-of-helsinki/. 19886379

