# Supplementary material for: Nursing master students’ experiences of and reflections on patient safety issues – a mixed-methods study using ecological momentary assessment
Source: BMC Med Educ. 2025 Nov 22;25:1744. doi: 10.1186/s12909-025-08281-6 (PMC12750830; doi:10.1186/s12909-025-08281-6)
Supplement: Supplementary file 1 — Supplementary Material 1. [file 12909_2025_8281_MOESM1_ESM.docx]

**Supplemental 1.** Interview Guide - Patient safety issues and utilization of the EMA Application

| 1. What are your experiences and thoughts on patient safety from the past? |
| --- |
| 1. How did you find the experience of using the mobile application? |
| 1. What have been positive experiences, and what have been less favourable experiences related to this learning situation? |
| 1. Have you gained any new insights pertaining to the topic of patient safety during your clinical practice thus far? |
| 1. Have you personally encountered any adverse events or near misses during your practice that could have had serious consequences for the patient? Based on this, are there topics that could be emphasized more in your education to prevent such incidents? |
| 1. What are your thoughts on teamwork in clinical practice? |
| 1. What do you find you learn the most from—the experiences that go well and function effectively, or those you wish had not occurred? Do you have any examples? |
| 1. Do you have any thoughts on why adverse events or near misses occur? Is this something that could have been prevented? If so, how? |
| 1. As the data collection period comes to an end, do you have any additional reflections on patient safety, adverse events, near misses, or positive occurrences where things have worked well that you would like to share? |
