# Supplementary material for: Nursing master students’ experiences of and reflections on patient safety issues – a mixed-methods study using ecological momentary assessment
Source: BMC Med Educ. 2025 Nov 22;25:1744. doi: 10.1186/s12909-025-08281-6 (PMC12750830; doi:10.1186/s12909-025-08281-6)
Supplement: Supplementary file 2 — Supplementary Material 2. [file 12909_2025_8281_MOESM2_ESM.docx]

**Supplementary Table 1.** Mean responses to the EMA statements and covariation with demographics

| **Statements** | | **1. Observed**  Mean (SD) | **2. Discussed**  Mean (SD) | **3. Avoided**  Mean (SD) | **4. Experienced**  Mean (SD) | **5. Safe work** Mean (SD) | **6. Threatened PS** Mean (SD) |
| --- | --- | --- | --- | --- | --- | --- | --- |
| **Overall** | | 2.5 (1.6) | 3.9 (1.4) | 2.5 (1.6) | 2.0 (1.4) | 4.6 (0.9) | 1.4 (0.8) |
| **Characteristics** | |  |  |  |  |  |  |
| **Age** | |  |  |  |  |  |  |
|  | <30 years | 2.4 (1.6) | 3.8 (1.4) | 2.7 (1.6) | 1.9 (1.4) | 4.7 (0.8) | 1.4 (0.7) |
|  | 30-39 years | 2.8 (1.7) | 4.1 (1.4) | 2.1 (1.5) | 2.1 (1.4) | 4.4 (1.1) | 1.5 (1.0) |
|  | 40-49 years | 1.8 (1.3) | 4.2 (1.3) | 2.7 (1.8) | 2.1 (1.5) | 5.0 (0.0) | 1.2 (0.4) |
| **Sex** | |  |  |  |  |  |  |
|  | Male | 2.6 (1.6) | 4.2 (1.2) | 2.6 (1.6) | 2.3 (1.5) | 4.7 (0.6) | 1.6 (1.0) |
|  | Female | 2.5 (1.7) | 3.8 (1.5) | 2.4 (1.6) | 1.8 (1.3) | 4.5 (1.0) | 1.3 (0.6) |
| **Specialty** | |  |  |  |  |  |  |
|  | NA | 2.3 (1.6) | 4.1 (1.3) | 2.5 (1.6) | 1.9 (1.4) | 4.7 (0.7) | 1.3 (0.8) |
|  | ICN | 2.8 (1.9) | 3.5 (1.6) | 2.7 (1.8) | 2.1 (1.5) | 4.7 (0.7) | 1.5 (1.0) |
|  | ORN | 2.7 (1.5) | 3.7 (1.6) | 1.6 (1.0) | 1.9 (1.3) | 3.6 (1.5) | 1.3 (0.5) |
| **Experience** | |  |  |  |  |  |  |
|  | <2 years | 2.9 (1.9) | 4.3 (1.4) | 2.3 (1.8) | 2.3 (1.8) | 4.3 (1.0) | 1.8 (1.5) |
|  | 2-4 years | 2.5 (1.7) | 3.5 (1.5) | 2.7 (1.6) | 2.0 (1.4) | 4.5 (1.1) | 1.4 (0.7) |
|  | 5-9 years | 2.4 (1.6) | 4.2 (1.3) | 2.2 (1.5) | 1.8 (1.3) | 4.6 (0.7) | 1.4 (0.9) |
|  | >10 years | 2.4 (1.7) | 4.5 (1.1) | 2.6 (1.8) | 1.9 (1.5) | 5.0 (0.0) | 1.1 (0.4) |
| **Evening** | |  |  |  |  |  |  |
|  | Day | 2.4 (1.6) | 4.1 (1.3) | 2.4 (1.7) | 2.0 (1.4) | 4.7 (0.7) | 1.4 (0.9) |
|  | Evening | 2.7 (1.6) | 3.7 (1.5) | 2.6 (1.5) | 1.9 (1.3) | 4.5 (1.0) | 1.4 (0.7) |
| Abbreviation: SD, standard deviation  ^a^ Estimated by using linear mixed effects model with restricted maximum likelihood estimation and a random intercept for each individual.  NA: Nurse Anesthetist; CCN: critical care nurse; and ORN: operation room nurse  Items: 1. Observed an error; 2. Discussed patient safety issues; 3. Avoided an adverse event; 4. Experienced an error or near-miss; 5. Safe work for the patient, and 6. Work has threatened the patient safety.  Likert scales (1-5) were used ranging from 1= Totally disagree to 5= Totally agree. | | | | | | | |
